# Intelligent Tire Prototype in Longitudinal Slip Operating Conditions

**DOI:** 10.3390/s24092681

**Published:** 2024-04-23

**Authors:** Jennifer Bastiaan, Abhishek Chawan, Wookjin Eum, Khalil Alipour, Foroogh Rouhollahi, Mohammad Behroozi, Javad Baqersad

**Affiliations:** Mechanical Engineering Department, Kettering University, Flint, MI 48504, USA

**Keywords:** intelligent tires, strain measurement, finite element analysis, physical testing

## Abstract

With the recent advances in autonomous vehicles, there is an increasing need for sensors that can help monitor tire–road conditions and the forces that are applied to the tire. The footprint area of a tire that makes direct contact with the road surface, known as the contact patch, is a key parameter for determining a vehicle’s effectiveness in accelerating, braking, and steering at various velocities. Road unevenness from features such as potholes and cracks results in large fluctuations in the contact patch surface area. Such conditions can eventually require the driver to perform driving maneuvers unorthodox to normal traffic patterns, such as excessive pedal depressions or large steering inputs, which can escalate to hazards such as the loss of control or impact. The integration of sensors into the inner liner of a tire has proven to be a promising method for extracting real-time tire-to-road contact patch interface data. In this research, a tire model is developed using Abaqus/CAE and analyzed using Abaqus/Explicit to study the nonlinear behavior of a rolling tire. Strain variations are investigated at the contact patch in three major longitudinal slip driving scenarios, including acceleration, braking, and free-rolling. Multiple vertical loading conditions on the tire are applied and studied. An intelligent tire prototype called KU-iTire is developed and tested to validate the strain results obtained from the simulations. Similar operating and loading conditions are applied to the physical prototype and the simulation model such that valid comparisons can be made. The experimental investigation focuses on the effectiveness of providing usable and reliable tire-to-road contact patch strain variation data under several longitudinal slip operating conditions. In this research, a correlation between FEA and experimental testing was observed between strain shape for free-rolling, acceleration, and braking conditions. A relationship between peak longitudinal strain and vertical load in free-rolling driving conditions was also observed and a correlation was observed between FEA and physical testing.

## 1. Introduction

Understanding the dynamics at the tire contact patch is crucial for analyzing ground vehicle performance. There have been many studies in the field of vehicle and tire dynamics for understanding tire–road interactions, specifically to analyze the handling and ride quality characteristics of vehicles in multibody dynamics software. In simulation and in the physical world, there are limits to the longitudinal and lateral forces that can be applied to the contact patch of each tire. These forces are governed by the coefficient of friction at the tire–road interface and the vertical force acting on the tire. Real-time availability of the governing parameters can largely help in estimating the fraction of Newtonian forces expended. Thus, it is very desirable to estimate these forces to prevent unsafe vehicle behavior.

For direct estimation of tire–road contact parameters, engineers are using sensor-based tires called ‘smart tires’ or ‘intelligent tires’. This new generation of intelligent tires could result in the development of novel vehicle control strategies based on direct information from the tire–road contact patch. Many studies focused on the concept of intelligent tires have attempted to characterize the contact parameters based on the type of sensor used and variables measured as features for estimation. In most cases, the idea is to acquire values related to tire dynamics such as Newtonian forces, tire slips, and friction characteristics from tire deformations. These deformations can be obtained by measuring quantities such as displacement, strain, or acceleration from the tire of a moving vehicle.

### 1.1. Review of Published Work

Efforts from industrial programs such as the APOLLO and FRICTION projects [1] have contributed to the understanding of strain energy extraction from the inner liner of a tire and the creation of an intelligent tire designed around increased traffic safety. Van Den Ende et al. [2] advanced the strain-based approach by harvesting and analyzing tire strain energy using piezoelectric deformation transducers. Braghin et al. [3] presented the idea of estimating several fundamental tire quantities such as longitudinal and lateral tire slip. These dynamic conditions were described by means of an accelerometer fixed inside the tire inner liner. Khaleghian et al. [4,5,6] employed a similar setup with a tri-axial accelerometer supported by a soft computing algorithm. This arrangement enabled the classification of terrain by identifying differences in the features of the experimental data. Goos et al. [7] developed a flexible ring tire model-based state estimator to predict the vertical load at the contact patch by employing a tri-axial accelerometer. An extended Kalman filter was used to predict the longitudinal and lateral tire forces.

There have been numerous physical testing studies of tires embedded with sensors on the sidewall as well as the inner liner. Pohl et al. [8] used Surface Acoustic Wave (SAW) sensors to monitor tire strain and estimate the tire–road friction coefficient. Lee et al. [9] also used machine learning for monitoring wheels for railway vehicles. Bastiaan [10] used piezoelectric deformation sensors, in concert with an artificial neural network, to estimate tire forces developed at the contact patch. Ergen et al. [11] and Savaresi et al. [12] used accelerometers to estimate tire forces. Gupta et al. [13] used an accelerometer to identify different road surface conditions. Kim et al. [14] developed a polynomial-based tire model by using a strain measurement method for an intelligent tire system. The relationship between tire strain data and vehicle driving conditions led to the development of a model comprising polynomial equations.

Kubba et al. [15] and other researchers such as Hall et al. [16] and Matsuzaki et al. [17] strongly support the use of Finite Element Analysis (FEA) to calculate tire deformation. Eder et al. [18,19] created a validated tire FEA model that was used to calculate tire deformation for a subsequent computational fluid dynamics analysis of an open-wheel race car when performing vehicle dynamics maneuvers. Zou et al. [20] used a tire FEA model in combination with a tire brush model to estimate the tire–road friction coefficient. The Abaqus FEA package has been at the forefront when it comes to predicting the elastically nonlinear material deformations of tire rubber in these studies. The feasibility of a tire physical experiment can be deduced from the results obtained from a tire FEA simulation.

The usefulness of tire FEA models in intelligent tire development requires that physical testing methods for the validation of these models exist. Some of the most practical methods for the physical validation of tire FEA models involve comparisons of dynamic stiffness and deformation behavior. For example, Patil et al. [21] performed a physical modal analysis of a tire to investigate the effect of inflation pressures on tire natural frequencies and mode shapes. This study found that tire natural frequencies are significantly influenced by inflation pressures, with an approximately linear relationship between inflation pressure and natural frequency. Such physically observed characteristics can be compared with similar predictions from tire FEA simulations.

Mange et al. [22] measured tire operating deflection shapes using a Digital Image Correlation (DIC) system. High-speed cameras were used to measure tire sidewall vibrations of a rolling racing tire that was mounted on a vehicle in a chassis dynamometer environment. Similar to traditional modal analysis, tire deformations obtained from DIC can be used to confirm correct behavior in tire FEA models. Matsubara et al. [23] measured contact patch strain contours for a tire rotating at 50 KPH using a photographic method. Strain contours measured from this physical testing method can provide valuable insight into the accuracy of a tire FEA model. Mousavi and Sandu [24] conducted a physical testing study of tires on ice with differing rubber stiffness. Tires with lower elastic moduli were found to have higher traction in icy road conditions. Insights such as these can be used to confirm the material models and coefficients used to represent rubber in tire FEA models.

Yunta et al. [25] used a strain-based method to identify tire tread deformation experimentally with sensors, where tests were carried out under different working conditions such as changing vertical loads and lateral tire slip angles. Both virtual and physical prototypes were studied in an attempt to observe any correlation or contradiction between the two methods. In general, understanding the similarities and differences in the results from simulation and experimental methods can provide enlightenment to those working in the Noise, Vibration, and Harshness (NVH) and tire dynamics fields.

### 1.2. Motivation for Current Work

The current work aims to identify critical features of tire strain signals that can be used for tire state estimation. Modern vehicles employ real-time feedback control for the implementation of safety systems such as electronic stability programs, anti-lock braking systems, and torque vectoring strategies [26]. However, such systems use data obtained from wheel speed sensors, accelerometers, and inertial measurement units to implement their respective control algorithms, thus indirectly estimating the tire–road contact parameters for control applications. For example, longitudinal tire slip may be determined according to wheel speed values, from which slip may be controlled. One concern with this type of control system is in its vulnerability to sudden changes in road conditions. The solution to this problem could be to find a direct means of estimating the tire–road contact parameters (such as tire forces, tire slips, and friction) in real time. This could ultimately result in new control algorithms that are designed to better contend with sudden changes in the tire–road interface.

The current paper proposes using strain energy as a reliable parameter for tire state estimators and providing an understating of change in strain energy for a tire during contact. The work uses both simulation and physical testing to validate this theory. Considering published work, there is not enough research in validating the findings from tire FEA studies with those from experimental tests. There is room for more published work in this area, and that is the primary contribution of this study. Here, a tire FEA model is used to understand the variation in tire inner liner longitudinal strain under pure longitudinal slip operating conditions, including acceleration, braking, and free-rolling. The vertical load on the tire is varied to observe the behavior of strain data, for the purpose of extracting features that can ultimately be used in vehicle control algorithms. Additionally, a tire physical prototype called KU-iTire is developed and equipped with strain gauges on the inner liner of the tire at the tread area, to support the findings of the FEA simulations.

This paper is organized as follows. The next section describes the tire FEA model. Section 3 discusses the physical testing. Results are shown in Section 4, and the conclusions are presented in Section 5.

## 2. Tire Finite Element Analysis Simulation

With the advancement of computational capabilities and the increased cost of developing physical prototypes for testing, researchers have increasingly been conducting simulated tests in virtual environments. Numerical simulation methods such as FEA are being used to investigate many practical structures, including tires [27,28,29,30,31]. FEA is a useful tool that not only replicates testing scenarios in a virtual environment but also provides the flexibility to perform design optimization at a low cost. FEA allows multiple design iterations to be investigated before time and money are spent on the production of prototype components. It can be used to calculate the stiffness, strength, and durability of structures, along with a prediction of their failure modes. The ability to predict the location and nature of a durability failure is vital in components such as tires, which may not be tested for failure in every conceivable service environment in advance of production.

### 2.1. Tire Representation

A tire is a complicated product made from diverse materials built around an elaborate structure. The major components of a tire are a high-stiffness cord and low-stiffness rubber. The cord generally consists of nylon and polyester yarns, especially at the sidewall location in automobile tires, whereas the tread areas are typically reinforced with steel cords. Tire cords are used as reinforcing materials. They aid in supporting the weight of a vehicle. The variety of reinforcing cords used inside the tire contributes heavily to the overall performance of the tire. Cords can vary in material, diameter, spacing, and orientation with respect to the circumferential direction of the tire. The tire size, type, load carrying requirements, and service inflation pressures are factors that determine cord material and geometry.

The tire FEA model for this research was originally created by Behroozi et al. [32]. It includes representations of tire rubber and internal tire reinforcements. The bonding of reinforcements with tire rubber is achieved using the rebar element function available in Abaqus. Rebar is the preferred method for defining reinforcements inside the host—solid rubber elements in Abaqus. To create the tire model, Behroozi et al. [32] had to physically cut a cross-section of the available tire and study and analyze every layer in the tire geometry. Rubber is known to exhibit highly nonlinear behavior under varying loads, including nonlinear elastic behaviors at very low deformation levels. Therefore, tire rubber is modeled as a Yeoh hyperelastic material in Abaqus. The ultimate goal of the tire FEA simulation is to produce usable strain data that will serve as a basis for determining the feasibility of an intelligent tire and generate input data for a control algorithm. On the other hand, the test represents an FSAE race car tire, not a passenger vehicle tire. The FSAE car tire model was selected to be used in the current research since the goal was to determine the feasibility of developing an intelligent tire and examine if the test and simulation could provide similar trends.

**Figure 1:**
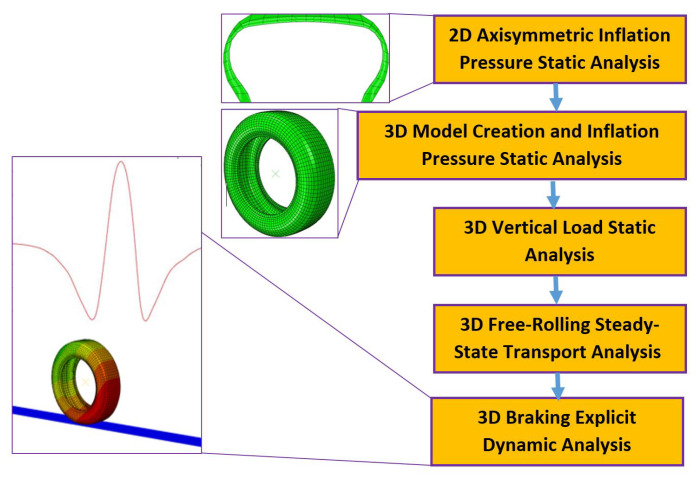
Flowchart of tire FEA simulation steps in Abaqus.

Contact between the tire and the road is particularly challenging to analyze when the traditional Lagrangian analysis approach is adopted for use with the finite element model. In that case, the model iterates through large numbers of increments and often suffers from convergence problems when the analysis is executed. One possible solution is to use a fine mesh at the contact area. When using an Abaqus/CAE 2021 analysis, refining the mesh in and around contact areas is recommended in order to increase the accuracy of the solution and to reduce the likelihood of numerical problems. In this work, a five-step FEA approach is adopted to make the model more accurate and computationally efficient, with a reduction in time consumed for each analysis iteration. For formulating slip, finite sliding was used between the tire contact patch and the road interface. Finite sliding was chosen since relative sliding between surfaces in contact was not to be ignored. Furthermore, slip conditions were integrated into the model for free-rolling application. Figure 1 is a flowchart depicting the five FEA steps used to predict virtual strain data from the tire FEA analysis.

**Figure 2:**
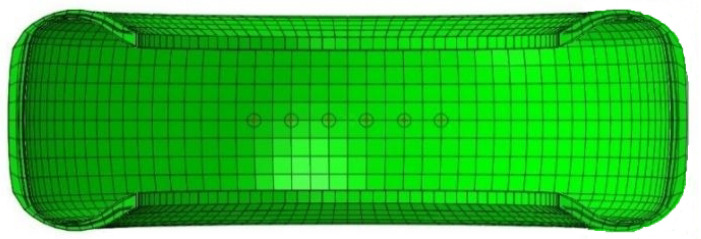
A cutaway view of the 3D tire FEA model showing virtual sensors at the inner liner (circled).

1. Two-dimensional axisymmetric inflation pressure analysis. The first step involves the creation and analysis of an axisymmetric two-dimensional (2D) cross-section model of the tire with an inflation pressure of 0.2 MPa (29 psi) applied at the tire inner liner. This *STATIC analysis, performed in Abaqus/CAE 2021, represents a typical tire inflation pressure loading scenario for automobile tires. The 2D tire model contains elements representing tire rubber, along with embedded ‘surface’ elements that carry the rebar cord reinforcements.
2. Three-dimensional model creation and inflation pressure analysis. In the second step, which is a *STATIC analysis performed in Abaqus/CAE 2021, the 2D cross-section model is revolved and extruded to generate a three-dimensional (3D) model of the tire. A 3D model of the tire is formed that includes hexahedral elements for representing the rubber parts of the tire, with embedded surfaces that stand for the tire reinforcements. The 3D tire model is loaded with a 0.2 MPa (29 psi) inflation pressure at the inner liner. Virtual sensors are created on the inner liner of the 3D tire model by requesting outputs for displacement, strain, and stress at specific elements and node sets, as shown in Figure 2. This approach helps in reducing the total computational time and storage space required for the analysis, as the desired variables are output only at defined nodes and elements, rather than everywhere in the model.
3. Three-dimensional vertical load analysis. The third step comprises a vertical load application at the reference point of the tire, which is at its center. This is a *STATIC analysis in Abaqus/CAE 2021 that puts the tire FEA model into contact with a rigid road surface. The vertical load applied to the tire model varied from 2000 N (450 lb) to 5000 N (1124 lb) in increments of 500 N (112 lb). Each vertical load was applied in a separate analysis step.
4. Three-dimensional free-rolling analysis. The fourth step is a straight line free-rolling analysis in Abaqus/CAE 2021. A *STEADY-STATE TRANSPORT analysis is performed. In this analysis method, the overall tire geometry is seen by a moving observer as defined by a set of points that are not moving. However, the material is observed to be moving with respect to the points that define the geometry. Thus, the global rigid body motion of the structure is not analyzed, but the local relative material deformation of the structure during motion is calculated. The translational velocity of the tire in the free-rolling analysis was 8 KPH (5 MPH). This translational velocity was selected to initiate a baseline for comparison with physical tire testing following the simulation work.
5. Three-dimensional braking analysis. The fifth step is a transient dynamic analysis of longitudinal tire motion in Abaqus/Explicit. In this fully dynamic analysis of type *DYNAMIC, EXPLICIT, the results from the fourth step for the steady-state rolling analysis in Abaqus/CAE 2021 are imported into Abaqus/Explicit. The import occurs at the start of the transient dynamic analysis. The rigid body rotating motion of the tire is fully analyzed in the fifth step, which is computationally expensive compared to the previous four analysis procedures.

### 2.2. Virtual Strain Data Extraction

**Figure 3:**
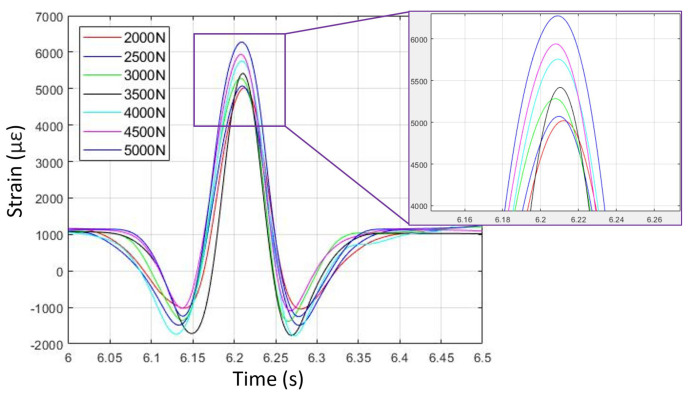
Free-rolling longitudinal strain versus time for seven vertical loads (from FEA simulation).

The focus here is to examine the variation in simulated longitudinal strain data during different longitudinal slip tire operating conditions, including free-rolling and braking. The longitudinal strain for one complete revolution of the tire FEA model is presented in the plots. Figure 3 shows virtual strain data versus time in free-rolling for seven different vertical loads. Generally, it can be seen from Figure 3 that the tire FEA model is predicting longitudinal strain levels in the tire tread region that are probably too high. Peak positive (i.e., tensile) strain levels, as predicted by the model, are around 6000 μϵ, or about 6%. Typically, such high strain levels would be expected in the sidewall region of the tire, which is less stiff than the tread region [33]. Therefore, the tire FEA model as developed should be used to investigate trends in longitudinal strain data in the tire tread area, rather than predict absolute values.

**Figure 4:**
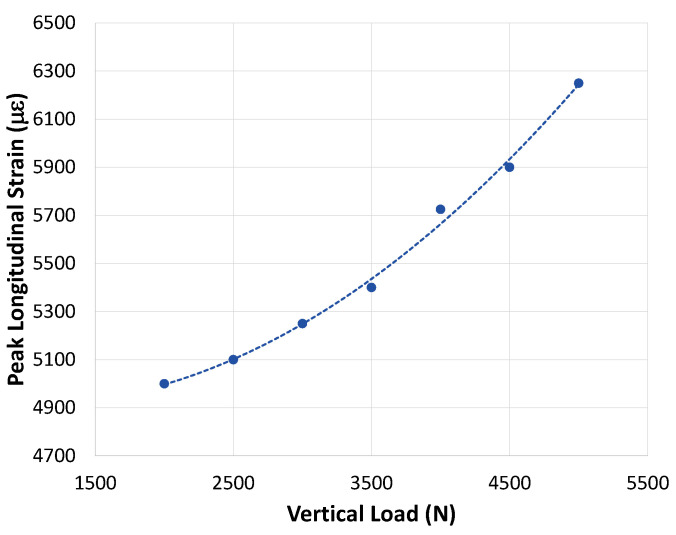
Relationship between peak longitudinal strain and vertical load in free-rolling (from FEA simulation).

A graph showing peak longitudinal strain in free-rolling versus vertical load is shown in Figure 4. It can be observed from Figure 4 that with an increase in vertical load, there is an increase in peak strain. In this case, the relationship between peak strain and vertical load can be described as a second-degree polynomial relationship with an $R^{2}$ value of 99.5%, indicating that the second-degree polynomial model is a good fit.

**Figure 5:**
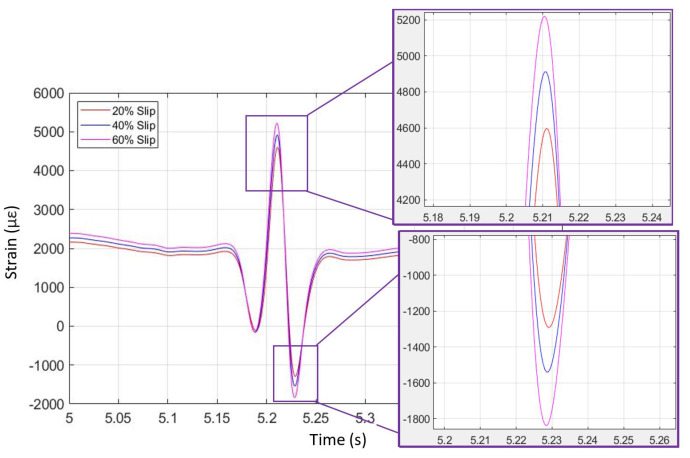
Braking longitudinal strain versus time for three longitudinal slip levels (from FEA simulation).

Figure 5 depicts simulated longitudinal strain data in braking, with three different levels of longitudinal slip analyzed. It can be observed that the slip variation contributes to deviations in the shape of the simulated strain curves. The free-rolling strain curves resemble a ‘Mexican hat’ formation. In the case of braking, the shape is distorted, whereby the trailing end of the ‘Mexican hat’ shape dips further into higher strain magnitudes. Note also that strain peak (maximum) and strain valley (minimum) values differ, depending on longitudinal slip level. Higher longitudinal slip levels result in higher maximum and lower minimum strain values. Furthermore, the difference in longitudinal slip level can be identified outside of the contact patch region. This can be seen at the start time of the plot in Figure 5, where a vertical offset can be seen that is associated with longitudinal slip. Higher longitudinal slip results in higher longitudinal strain, outside of the contact patch.

## 3. Intelligent Tire Physical Testing

**Figure 6:**
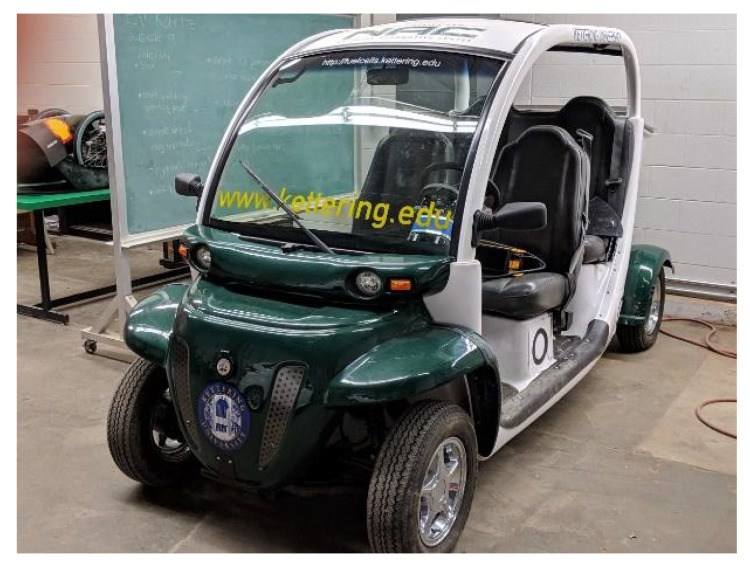
The 2003 Polaris GEM test vehicle.

The fabrication of the KU-iTire prototype began following the completion of the tire FEA study, to further investigate the feasibility of an intelligent tire. The vehicle employed during prototype tire testing was a 2003 Polaris Global Electric Motorcar (GEM), as shown in Figure 6.

### 3.1. Prototype Fabrication

**Figure 7:**
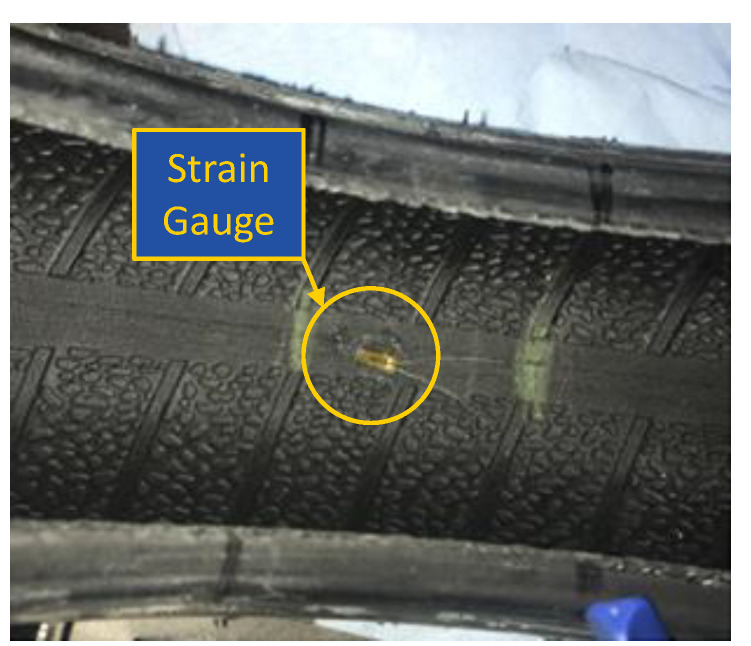
Strain gauge installed on the tire inner liner at the tread centerline.

**Figure 8:**
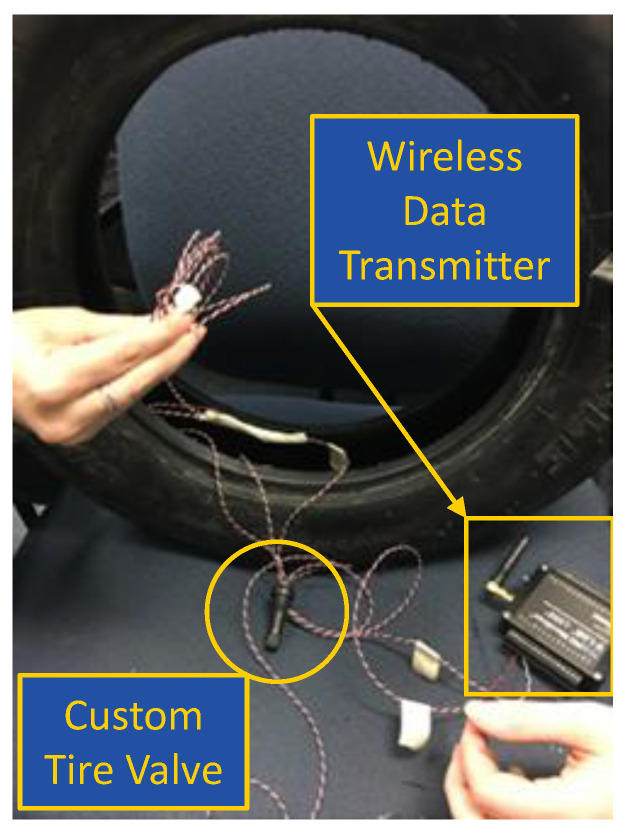
Strain gauge wires, customized tire valve, and wireless data transmitter.

A tire of size 185/80R13 was used to develop the KU-iTire prototype. The tire had a load index of 82 and nylon plies. The tire was mounted on a steel wheel with five lug holes. A strain gauge was installed at the inner liner of the tire, oriented parallel with the tire tread, as shown in Figure 7. The strain gauge was attached to the tire inner liner with a specialized resin from the strain gauge manufacturer. The strain gauge lead wires were soldered to connecting wires for data transmission. Wires from the strain gauge were passed to the outside of the tire through a customized tire valve, as shown in Figure 8.

Selecting the strain gauges was challenging, since most strain gauges have low strain measurement limits. For the KU-iTire application, HBM LY11 microstrain gauges capable of measuring high strains, up to 200,000 μϵ, were used. For data acquisition, a LORD MicroStrain Sensing System was used. This wireless data acquisition system consisted of two units, a transmitter and a base unit. The base unit communicated with a research computer to store strain data. Node Commander 2.14.0, the software suite proprietary to LORD, was installed in the computer that served as the storage location for the strain data from the base station.

**Figure 9:**
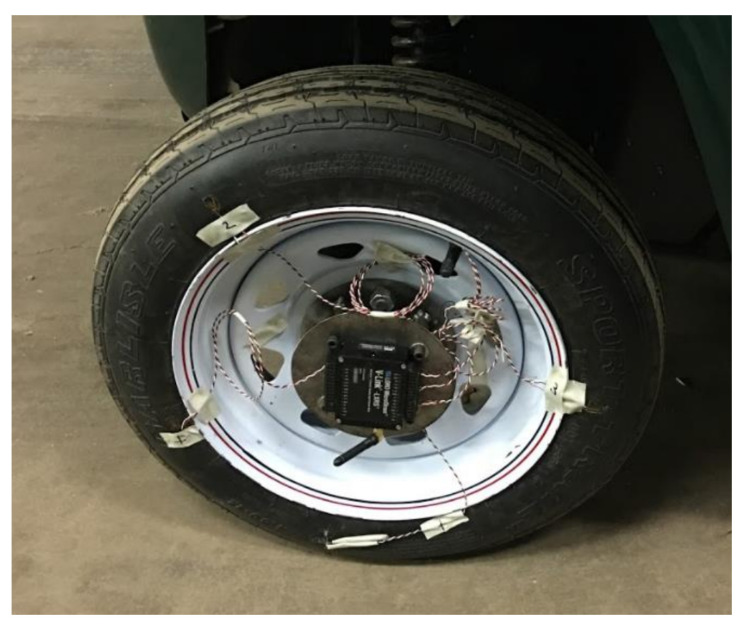
KU-iTire prototype with wireless strain data transmitter at center.

The wireless data transmitter was mounted on a specially designed steel mounting structure, which was itself attached using the original equipment wheel hub bolts. Damping material was added between the steel mounting structure and the wireless data transmitter, to reduce vibration experienced by the transmitter that could distort the strain signals. A photograph of the finished KU-iTire prototype appears in Figure 9. This prototype was installed at the left front corner of the GEM vehicle.

### 3.2. Test Setup and Conditions

**Figure 10:**
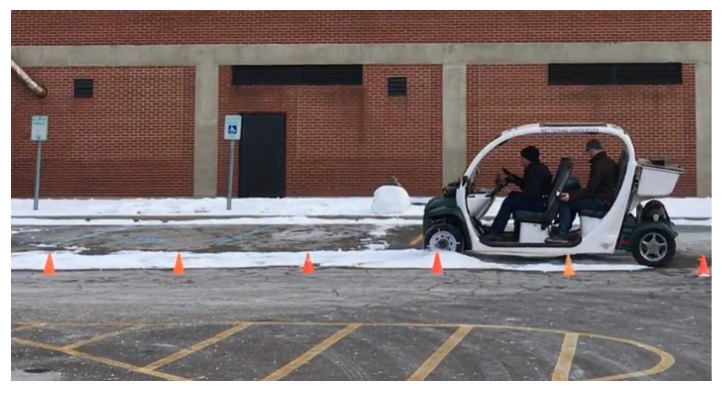
Parking lot physical testing of KU-iTire prototype.

**Figure 11:**
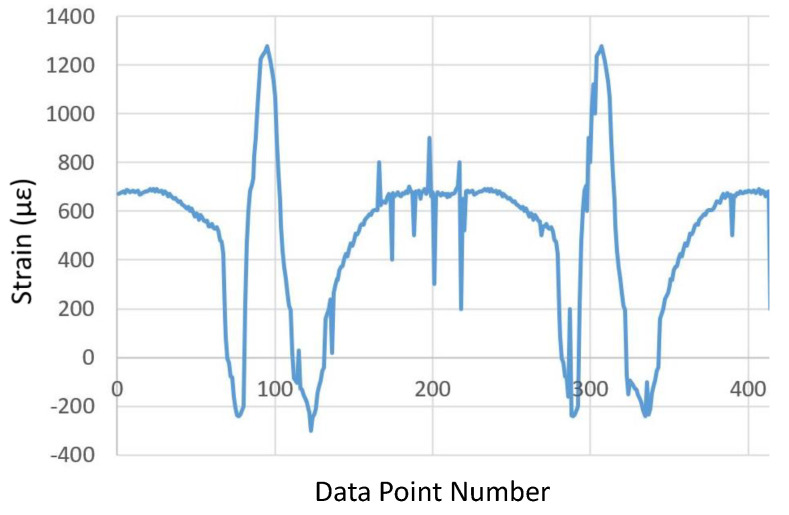
Free-rolling longitudinal strain versus data point number (raw strain data from physical test).

The physical test was designed to equip the GEM vehicle with the KU-iTire prototype and drive the vehicle on paved roads to capture real-world data. However, since the durability of the strain gauges and their attachment adhesive was a concern, the testing was conducted in a controlled setting in a parking lot, as shown in Figure 10. Physical testing was performed under dry and snowy weather conditions. Figure 11 is a plot of free-rolling longitudinal strain acquired directly from the data acquisition system, where two complete revolutions of the left front wheel are shown. In this study, the physically measured data were post-processed using moving average filters in MATLAB to eliminate the measurement noise and short-term fluctuations that can be seen in Figure 11.

*Test operating conditions.* A sample size of six test runs for each operating condition was recorded. The operating conditions considered for the test were as follows:

1. Free-rolling at 8 KPH (5 MPH) constant speed with *two* passengers on dry pavement.
2. Free-rolling at 8 KPH (5 MPH) constant speed with *three* passengers on dry pavement.
  - Representing an approximately 10% increase in front axle load compared to the first condition.
3. Free-rolling at 8 KPH (5 MPH) constant speed with *four* passengers on dry pavement.
  - Representing an approximately 20% increase in front axle load compared to the first condition.
4. Braking from 19 KPH (12 MPH) to stop on dry pavement.
5. Braking from 19 KPH (12 MPH) to stop on snow-covered pavement.
6. Acceleration from 5 KPH (3 MPH) to 29 KPH (18 MPH) on snow-covered pavement.

An 8 KPH (5 MPH) constant vehicle speed was used in conditions one to three to match the tire FEA simulation, where this same translational velocity was studied. Otherwise, conditions one to three were selected to investigate how differing vertical loads can alter the physically measured strain data, as a way of confirming the virtual strain data. The virtual strain data were altered when the tire was subjected to different vertical loads. For the braking and acceleration tests of conditions four through six, the mechanical limits of the GEM test vehicle were the limiting factors for determining vehicle test velocities.

## 4. Strain Data Comparison between Simulation and Physical Testing

To engender confidence in the tire FEA simulation results, physical prototype testing was executed, focusing on the observation of features that were identified in the simulation data. The shapes of the strain curves were of particular interest.

### 4.1. Free-Rolling

**Figure 12:**
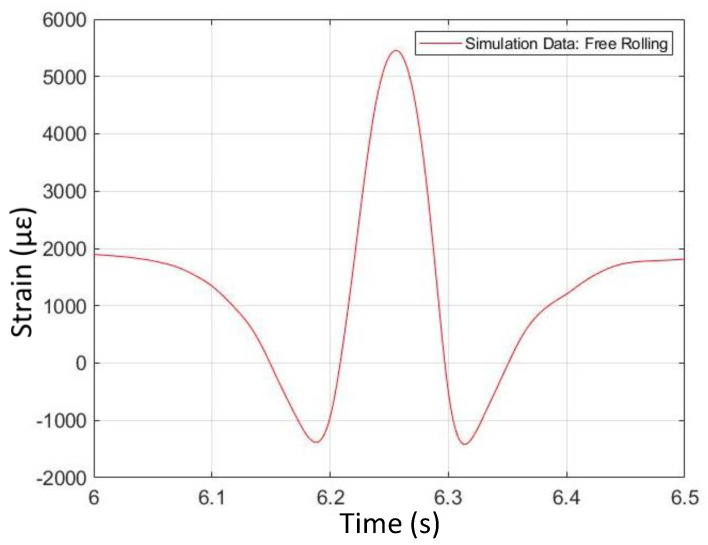
Free-rolling longitudinal strain versus time (from FEA simulation).

**Figure 13:**
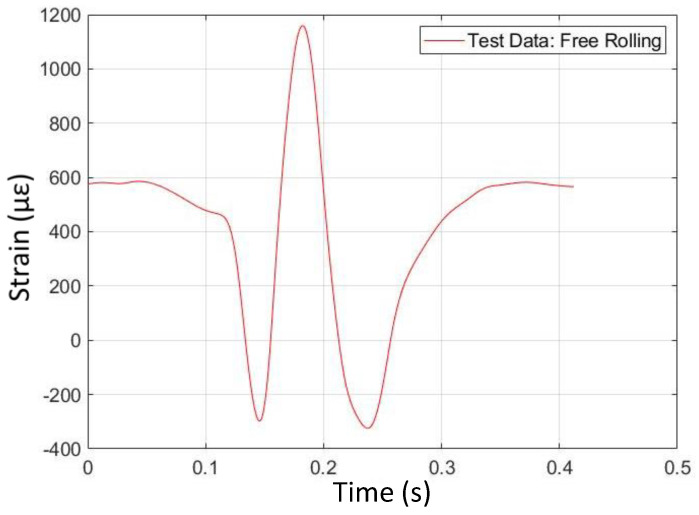
Free -rolling longitudinal strain versus time (from physical test).

Figure 12 and Figure 13 illustrate the shape comparison between simulation data and physical test data for free-rolling conditions on dry tarmac with two passengers (first operating condition). The simulation strain data have a ‘Mexican hat’ shape, with a positive (tensile) strain peak near the center of the contact patch and two strain valleys. This curve is approximately symmetrical about the center of the contact patch. The shape of strain data from the physical test corroborates the simulation data, where the physically measured strain curve has a similar hat shape compared to the simulation strain curve.

Interestingly, the physically measured strain curve is not as symmetrical about the contact patch center, in comparison with the simulated curve. Also, note that the strain levels, as predicted by the simulation, are much higher than the physically measured strain levels, up to five times higher at some points in the tire rotation cycle. Based on previous work, it is likely that the strain levels from the physical test are reasonable, with around 1% peak longitudinal strain at the center of the contact patch in the free-rolling condition. As the tire FEA model represents a different tire than the one that was physically tested, however, a comparison of the strain levels is less important than a comparison of the strain curve shapes.

### 4.2. Braking

**Figure 14:**
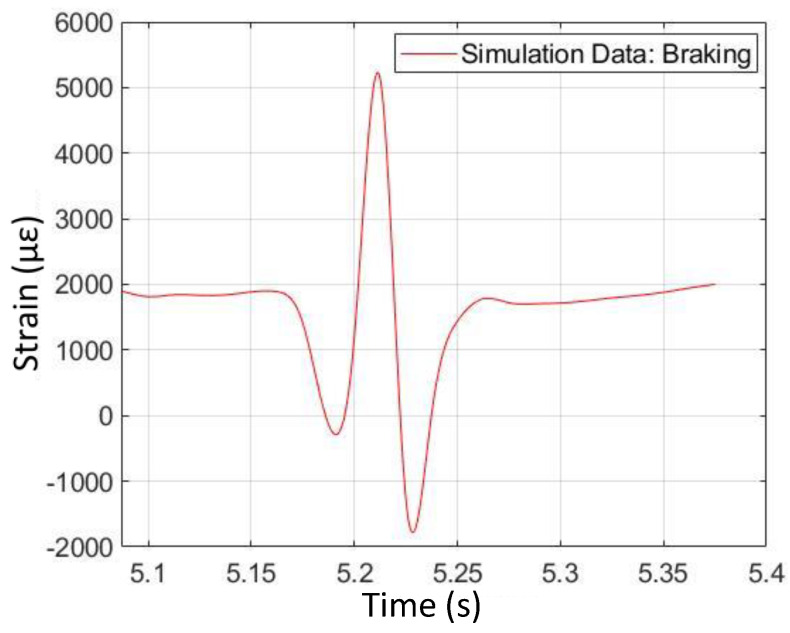
Braking longitudinal strain versus time (from FEA simulation).

**Figure 15:**
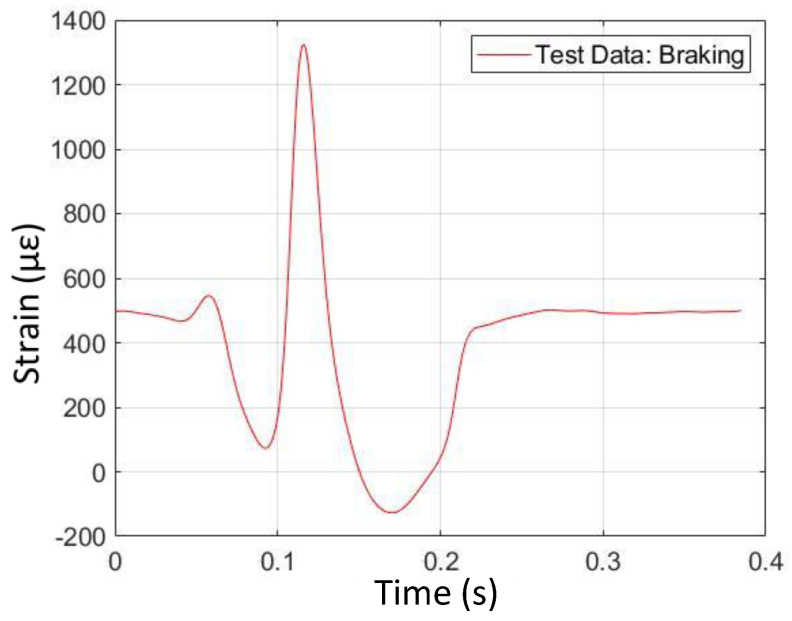
Braking longitudinal strain versus time (from physical test).

Plots of strain data from simulation and physical testing from braking (fourth operating condition) appear in Figure 14 and Figure 15. From these plots, it can be seen that the characteristic shape associated with braking, as opposed to free-rolling, is asymmetric in the longitudinal strain curve. The magnitude of the second strain valley at the rear of the contact patch is greater than that of the first strain valley at the front of the contact patch. Both the simulation data and the physical test data exhibit this behavior. From Figure 15, it can be seen that the physically measured longitudinal strain at the bottom of the second valley is negative (i.e., compressive) strain. This is in contrast with the bottom of the first valley, where the strain is positive (i.e., tensile). In general, the physical test data confirm the simulation data with respect to the trends in braking. However, there is an interesting difference in the strain curve shapes, where the second valley is extended in time in the physical test data. This feature is not observed in the simulation data.

### 4.3. Vertical Load Variations

**Figure 16:**
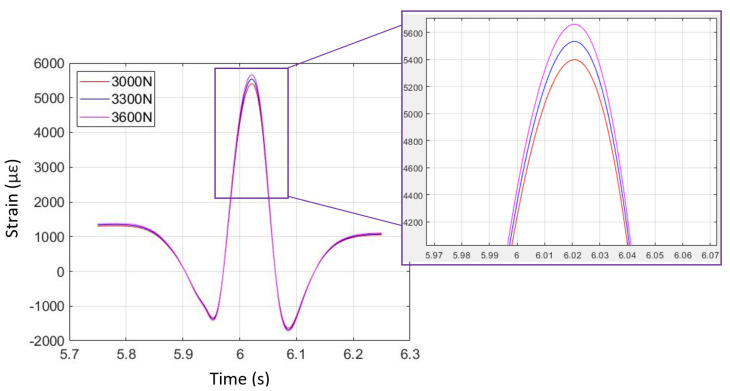
Free-rolling longitudinal strain versus time for three vertical loads (from FEA simulation).

**Figure 17:**
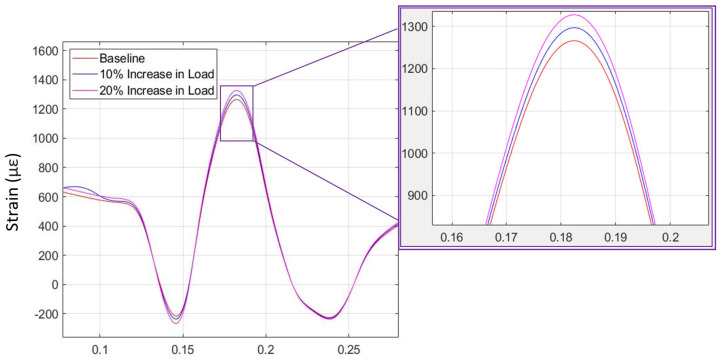
Free-rolling longitudinal strain versus time for three vertical loads (from physical test).

The strain data from simulation and physical testing for free-rolling on dry pavement (first to third operating conditions) are shown in Figure 16 and Figure 17. Three different vertical loads were applied to the KU-iTire prototype in the physical test, all in concert with the free-rolling operating condition. The tire FEA simulation was solved for the free-rolling case at three different vertical loads of 3000 N, 3300 N, and 3600 N, as these force levels were similar to the three different vehicle corner loads in the physical tests. Figure 16 and Figure 17 illustrate the strain curves for free-rolling with varying corner loads from both simulation and physical testing. As shown in the magnified insets, the difference in vertical load can be most clearly identified by the peak longitudinal strain level, with higher loads producing higher peak strain levels. This same behavior was observed in both the simulation strain data and the physical test strain data. In the case of the physical test data, a 10% increase in vertical load resulted in a 3% increase in peak strain level.

### 4.4. Differing Operating Conditions

**Figure 18:**
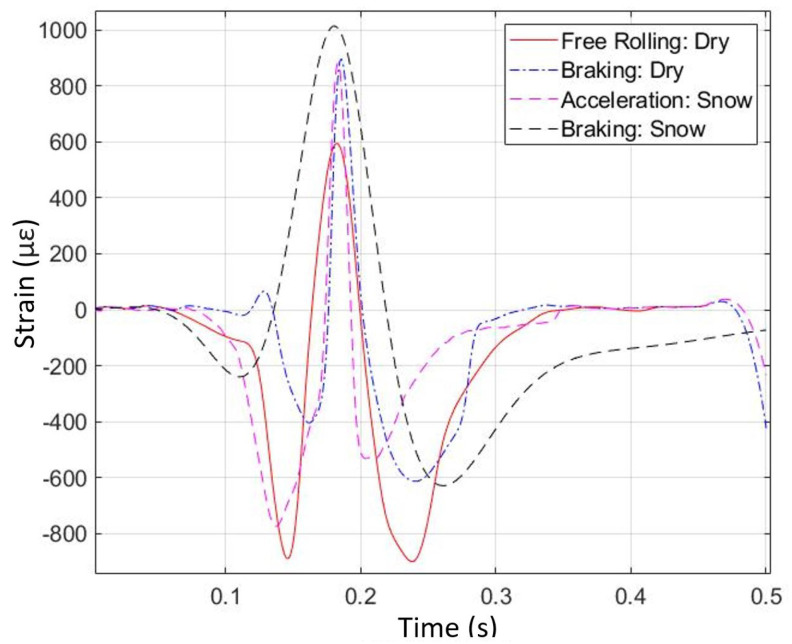
Longitudinal strain versus time for four different operating conditions (from physical test).

A plot showing longitudinal strain for four different operating conditions appears in Figure 18. In this plot, the strain levels outside the contact patch have been moved to the zero line, so the curves can be easily compared. In practice, the strain offset in the vertical axis of the plot (outside the contact patch) is associated with inflation pressure [34]. Here, the offsets have been subtracted to make up for minor differences in pressure. From the plot, it can be seen that the longitudinal strain curves are modified significantly by the different operating conditions.

For example, the snow braking curve (fifth operating condition) is significantly different than the dry braking curve (fourth operating condition). The strain peak is higher in the snow braking case, and the central peak is much wider. Additionally, the snow acceleration curve (sixth operating condition) is not exactly the mirror image of the snow braking curve (fifth operating condition), even though it could be assumed that this is the case in the absence of measured data. While some features are mirrored about the center of the contact patch, such as the deeper valley (at the front in the case of acceleration, at the rear in the case of braking), there are important differences such as the width of the peak, which is wider for braking. These results suggest that longitudinal strain measurements are a means of identifying both the vehicle dynamics maneuver being performed and the condition of the road being traversed.

## 5. Conclusions and Future Scope

The tire FEA model was able to predict the longitudinal strain of the tire over one wheel revolution for both free-rolling and braking. The shapes of the strain curves as predicted by the model were generally correct. However, the simulated strain curves were more symmetrical about the center of the contact patch compared to the physically measured curves. This discrepancy in the model should be investigated further. Moreover, the unreasonably large strain levels that were predicted by the model should be studied, with the goal of improving the accuracy of its predicted strain magnitudes. Once the model is improved, it will be a useful tool for predicting tire strain and developing the KU-iTire prototype.

Results from the tire FEA model show that there is a second-degree polynomial relationship between peak longitudinal strain and vertical load in free-rolling. This relationship can be used in a system that estimates tire vertical load from longitudinal strain measurements. Physical testing of longitudinal strain also showed a relationship between longitudinal strain and vertical load in free-rolling, with increasing vertical loads resulting in increasing peak strain levels. Not enough data points were collected to confirm the polynomial relationship of the tire FEA simulation, however. More physical testing should be performed to confirm the relationship.

This work concentrated on observing longitudinal strain variation in certain pure longitudinal slip operating conditions and post-processing the strain to identify features that can act as input parameters to a vehicle control algorithm. For example, it may be possible to use peak longitudinal strain at the tire inner liner to estimate tire vertical load. Additional strain features can be identified through further study of the KU-iTire prototype in other vehicle events, including pure lateral slip and combined slip maneuvers. A wide range of tire inflation pressures, vehicle velocities, and road friction conditions should be studied to identify tire strain characteristics that can be associated with tire and vehicle states, with the goal of enhancing ground vehicle performance and safety.

One of the limitations of the current study is the cost associated with strain gauges that can last for the life of a tire. Most conventional strain gauges cannot resist the deformations that tires experience.

## Data Availability

Data are contained within the article. For fruther information, please contact Javad Baqersad at jbaqersad@kettering.edu.
